# Usefulness of Lung Ultrasound in Paediatric Respiratory Diseases

**DOI:** 10.3390/diagnostics11101783

**Published:** 2021-09-28

**Authors:** Francesco Sansone, Marina Attanasi, Paola Di Filippo, Giuseppe Francesco Sferrazza Papa, Sabrina Di Pillo, Francesco Chiarelli

**Affiliations:** 1 Paediatric Allergy and Pulmonology Unit, Department of Paediatrics, University of Chieti-Pescara, 66100 Chieti, Italy; francesco.sansone001@alumni.unich.it (F.S.); marina_attanasi@hotmail.it (M.A.); difilippopaola@libero.it (P.D.F.); sabrinadipillo@gmail.com (S.D.P.); 2 Dipartimento di Scienze della Salute, Università degli Studi di Milano, 20146 Milan, Italy; francesco.sferrazza@gmail.com; 3 Department of Neurorehabilitation Sciences, Casa di Cura del Policlinico, 20144 Milan, Italy

**Keywords:** lung ultrasound, children, respiratory diseases

## Abstract

Respiratory infection diseases are among the major causes of morbidity and mortality in children. Diagnosis is focused on clinical presentation, yet signs and symptoms are not specific and there is a need for new non-radiating diagnostic tools. Among these, lung ultrasound (LUS) has recently been included in point-of-care protocols showing interesting results. In comparison to other imaging techniques, such as chest X-ray and computed tomography, ultrasonography does not use ionizing radiations. Therefore, it is particularly suitable for clinical follow-up of paediatric patients. LUS requires only 5–10 min and allows physicians to make quick decisions about the patient’s management. Nowadays, LUS has become an early diagnostic tool to detect pneumonia during the COVID-19 pandemic. In this narrative review, we show the most recent scientific literature about advantages and limits of LUS performance in children. Furthermore, we discuss the major paediatric indications separately, with a paragraph fully dedicated to COVID-19. Finally, we mention potential future perspectives about LUS application in paediatric respiratory diseases.

## 1. Introduction

Respiratory tract diseases remain a global challenge for paediatricians. Pulmonary infections are a major source of morbidity and mortality not only in developing countries, but also in the western world [1]. However, diagnostic and therapeutic approach to these pathologies has not changed dramatically in the last years. Indeed, paediatric guidelines are still relying mainly on clinical presentation and non-specific laboratory and imaging tests [2,3]. Among the latter, computed tomography (CT) is considered the gold standard for lung disease in the adults [4], yet it is not routinely performed in children, due to high ionizing radiation exposure [5,6,7]. The most used radiographical test is the chest X-ray (CXR), which is fast and standardized by World Health Organization (WHO) guidelines [8]. However, CXR is far from being considered the optimal diagnostic tool for lung assessment in children, as it implies a non-negligible radiation exposure, it has poor inter-operator reliability and it lacks sensitivity and specificity with respect to CT scans [9,10]. Recently, lung ultrasound (LUS) has gained more and more attention as a useful diagnostic tool in adults and in children, as well. Its application fields have expanded during the last years throughout different age spans and different pathologies [11], from pneumonia to rare congenital malformations. During the Coronavirus Disease 19 (COVID-19) pandemic in 2020, LUS has played a crucial role in screening affected individuals, pointing out patients requiring hospitalization [12].

In this narrative review we discuss the main application fields of LUS in childhood, with a focus on the advantages and limitations of this imaging technique. We conclude with a final paragraph about its present role in paediatric respiratory disease management and future perspectives.

## 2. Materials and Methods

**Figure 1:**
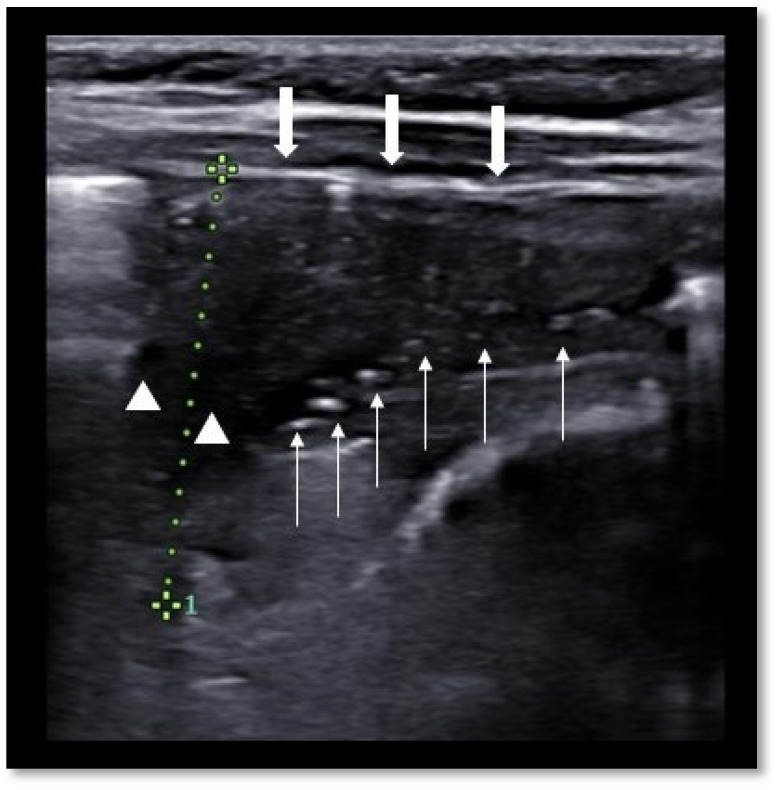
Basal lung consolidation in a 8 month-old girl, showing hypoechoic triangular shape, pleural line attenuation (thick arrows), air (thin arrows) and fluid (triangles) bronchograms. Depth is approximately 2.5 cm (dotted green line). Ultrasonographic appearance is compatible with pneumonia. Image captured using a 3.0–16.0 MHz linear array transducer.

We searched for articles on PubMed using the keywords “lung ultrasound”, “children”, “lung diseases”, and “respiratory diseases”. In particular, we used the following search terms and logic: “*lung ultrasound AND lung diseases OR respiratory diseases AND children OR childhood OR paediatric age OR infancy*”. Further studies were obtained through the references of some papers. Articles were selected according to their title and abstract, using eligibility criteria. The inclusion criteria were: being in the English language; paediatric study population (age range 0–18 years old); and type of study, including narrative and systematic reviews, longitudinal retrospective and prospective studies, and randomized control trials. Adult studies, case reports, expert opinions and manuscripts published in a language other than English were excluded. Papers published before 2016 were excluded. We selected 76 papers from a total of 1888 inherent articles (Figure 1). The final reference list was developed based on originality and relevance to the broader scope of this review.

## 3. LUS in Neonatal Diseases

The newborn shows peculiar ultrasonographic pulmonary patterns which can be used to rule out pathological conditions [13]. B-lines are the most common artifact in neonates and can be found even in physiologic conditions within 24 to 48 h after delivery [14]. B-lines gradually disappear as interstitial and alveolar fluids are resorbed. In neonates with smaller gestational age and caesarean delivery, B-lines can persist much longer [15]. Confluent B-lines, or white lung pattern, can be easily found in newborns due to a relatively higher percentage of fluids and a lower glomerular filtration rate with respect to older children [15]. Table 1 summarizes the neonatal lung disorders which can be assessed using LUS.

Two common neonatal pathological conditions are transient tachypnoea of the newborn (TTN) and respiratory distress syndrome (RDS). Both are characterized by dense confluent B-lines, representing different grades of pulmonary edema, and by alterations of the pleural line [16]. Specific LUS signs can help the neonatologist to differentiate between these two conditions. Several authors noted that TTN is characterized by spared lung areas and no consolidation, whereas RDS involves the entire lung bilaterally with interspersed consolidations [15,17]. The double-lung point (very compact B-lines in the inferior pulmonary fields and scattered B-lines in the superior ones [18]), once thought to be pathognomonic for TTN, has been recently demonstrated to be a non-specific sign [19,20]. Rachuri et al. [17] showed that LUS was more accurate in distinguishing neonatal respiratory distress aetiology with respect to CXR.

LUS showed high sensitivity for detection of meconium aspiration syndrome (MAS), characterized mainly by diffuse lung consolidations, pleural irregularities and B-lines [21]. These signs are not specific for MAS and could also be found in pneumonia and RDS [21]. 

Pulmonary malformations undetected by a prenatal ultrasound screening test can be found with LUS examination [15,22]. This is particularly true in low socio-economic contexts where prenatal assessment is not routinary performed. LUS can identify congenital diaphragmatic hernia and congenital pulmonary airway malformations (CPAMs), such as cystic lesions or consolidated tissue-like lesions [7,16,19,23]. The ultrasonographic test should be followed by a CT scan to measure the extension of the lesion [24].

LUS signs have also been used as a semi-quantitative tool to predict the need for surfactant therapy in patients affected by RDS, and the need for mechanical ventilation in newborns with clinical signs of respiratory distress and low oxygen saturation [24]. Brat et al. [25] created a LUS score (LUSS) system based on the presence of A-lines, normal pleural line, B-lines and consolidations, identifying three main aeriation patterns. The real accuracy and applicability of this score has yet to be established. In fact, many items need to be clarified, such as the cut-off for the pathological B-lines number, definition of focal lung edema, extent of consolidations and coexistence of both B-lines and consolidations [26]. Moreover, LUSS requires the assessment of all lung surfaces (i.e., anterior, lateral and posterior) which could be difficult to obtain in critically ill neonates [27]. Notwithstanding, few studies sustained that LUSS had a better sensitivity with respect to the clinical score in pointing out children requiring surfactant replacement, particularly in preterm newborns of <30 weeks of gestational age [28,29]. Perri et al. [30] demonstrated that LUSS could be used for the follow-up of newborns treated with surfactant replacement, monitoring the need for a second treatment after 2 h from the first administration.

Despite LUS’ methodological limits, the use of ultrasounds to assess neonates with pulmonary distress proved to be helpful in supporting clinical diagnosis and follow-up, mostly in Neonatal Intensive Care Units. Scored systems based on LUS findings might be used to predict the need for ventilatory support in the near future. However, larger prospective studies are needed to validate those scores.

## 4. LUS in Pneumonia

Pneumonia is one of the most common respiratory diseases of childhood [1]. Diagnosis can be challenging, as there are no specific signs and symptoms. Recent guidelines do not recommend radiological exams in children with suspected pneumonia [2,3]. CT is considered the gold standard to rule out pneumonia in adult patients, but it is rarely used in children due to high radiation exposure [5,6,7]. In the last years, LUS has been studied to test its efficacy in diagnosing pneumonia in children, with generally good results. When compared with chest X-ray and clinical scores, LUS showed good sensitivity and specificity [6,10,31,32,33,34,35,36,37,38,39]. Moreover, LUS seemed to have greater sensitivity and less specificity than CXR, suggesting its usefulness as a screening tool for pneumonia [6,10,40]. One of the major problems when assessing LUS’ diagnostic performance is the lack of an ideal reference standard. An Iranian prospective study matched LUS, CXR and CT results using CT as the reference standard [32]. The authors showed that LUS and CXR were not as accurate as CT in diagnosing uncomplicated pneumonia, whereas there was almost full agreement between LUS and CT in complicated pneumonia patients [32]. They concluded that the integration of LUS with CXR could be a reliable surrogate for CT scans in children [32]. Lissaman et al. [35] found both a higher sensitivity and specificity of LUS with respect to CXR, confirming its reliability as a screening tool [35]. They added that a negative LUS did not exclude the presence of a consolidation, as lesions hided by the mediastinum or bone structures and deep lesions not reaching the pleura could be missed by LUS [35]. Two recent meta-analyses collected the available literature about this issue [34,41]. The first, by Orso et al. [41], showed a good level of accuracy in diagnosing community acquired pneumonia (CAPs) compared to clinical presentation and CXR alone. The second, by Jun-Hong Yan et al. [34], showed no statistical difference of pooled sensitivity and specificity between LUS and CXR [34]. However, both meta-analyses found a high degree of heterogenicity among the several studies collected, which the authors attributed to a variable degree of experience among the involved physicians and different reference standards [34,41].

When detected by LUS, lung consolidations show many aspects, such as the overall echogenicity, dimensions, and presence of air or fluid bronchograms (Figure 1). In a prospective study, Berce et al. [42] aimed to discover ultrasonographic signs which could reliably differentiate bacterial CAP from viral and atypical CAP. They found that viral CAP showed more commonly bilateral involvement with multiple consolidations, whereas bacterial CAP was usually monolateral with a single consolidation [42]. There was a significant difference in consolidation size between viral and bacterial CAPs, the latter showing a major extension from the pleural line. The best extension cut-off calculated was 21 mm, with a sensitivity of 80% and a specificity of 75% [42]. In addition, bacterial consolidations showed a faster regression rate with respect to viral and atypical CAPs. Similar results were presented in another prospective cross-sectional study showing LUS’ high sensitivity and specificity for bacterial pneumonia and lower sensitivity for viral pneumonia [43].

Some ultrasonographic signs are difficult to interpret, such as the presence of multiple small subpleural consolidations with a maximum diameter <2 cm [31]. Those signs are not detected by CXR and their pathological meaning is not fully ascertained. Iorio et al. [33] studied the distribution of these small consolidations, which are called satellite lesions when a bigger lesion coexists. They hypothesized that satellite lesions homolateral to the main lesion could be suggestive of bacterial aetiology, whereas a contralateral lesion could be representative of an atypical or viral pneumonia caused by the lymphatic drainage [33].

**Figure 2:**
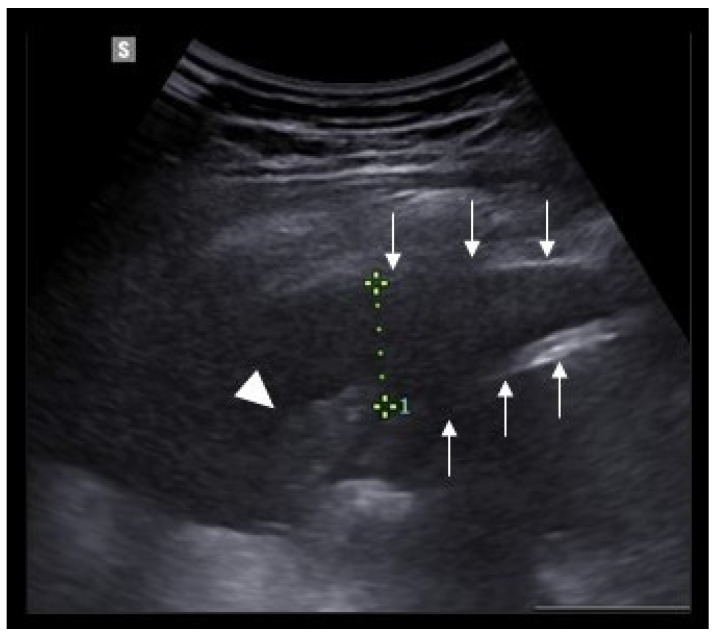
Pleural effusion in a 16-year-old boy, showing fine echoes, extending 2 cm (doted green line) from parietal pleura (down-pointing thin arrows) to the diaphragm (up-pointing thin arrows). An atelectatic compressed lung is visible at the bottom of the picture as a triangular hypoechoic formation (white triangle). Image captured using a 1.0–7.0 MHz curved array transducer.

Besides diagnosis, there is evidence supporting the employment of LUS to guide the management and follow-up of pneumonia patients. Ultrasonographic follow-up correlates well with the final outcome better than the clinical course or laboratory tests [44]. Musolino et al. [45] observed that patients who developed complicated pneumonia did not show signs of ultrasonographic improvement after 48 h of treatment. Pneumonia complications can be assessed using contrast-enhanced ultrasound (CEUS) [46]. Lung necrosis is characterized by non-enhanced areas of consolidation after intravenous injection of microbubble contrast. In addition, complicated parapneumonic effusion can be studied by directly injecting the contrast medium inside the pleural cavity through the draining catheter [46]. James et al. [47] adopted a LUS based grading system to guide fibrinolytic treatment in complicated parapneumonic effusion [47] (Figure 2). Prospective validation of this grading system would ameliorate fibrinolytic efficacy in these patients and reduce the risk of pleural bleeding [47].

LUS proved to be a valuable imaging technique to detect lung consolidations in pneumonia, despite its methodological limits. Sensitivity and specificity are tricky to assess, as there is not an ideal reference standard in children. On the basis of a single study [43], LUS sensitivity seems to be higher for bacterial CAPs than for viral ones. Current evidence supports the use of LUS in paediatric pneumonia treatment and follow-up and in the characterization of pleural effusions. Moreover, ultrasonographic characteristics of lung consolidations seem to distinguish CAP aetiology better than CXR. However, some findings are difficult to interpret, such as subpleural consolidations < 2 cm. Future studies will better investigate the role of LUS in pneumonia diagnosis.

## 5. LUS in Bronchiolitis, Pneumothorax and Other Paediatric Respiratory Diseases

Pathologic artifacts on LUS are not only useful for diagnosing pneumonia, but can point out a list of other lung diseases that should be taken into consideration in the appropriate clinical setting.

Bronchiolitis is one of the most common respiratory diseases in infants < 24 months of age [48]. It is caused by viral infections, mainly by Respiratory Syncytial Virus [49]. Diagnosis is based on clinical presentation, but distinguishing simple bronchiolitis from bacterial over-infection can be very challenging [50,51,52]. CXR is generally not helpful, as it cannot reliably classify radiopacities aetiology. In 2018, an Italian study group compared LUS versus CXR in detecting signs of pneumonia in children hospitalized for bronchiolitis [53]. LUS was more sensitive and even more specific than CXR when taking into consideration only consolidations > 1 cm [53]. Ingelse et al. [54] further explored the clinical utility of LUS in bronchiolitis affected patients, showing that a score based on LUS findings correlated with oxygenation anomaly on the first day of mechanical ventilation in severely affected children hospitalized in the PICU. The LUS score has also been used to predict the need for oxygen supplementation and its modality (high flow nasal canula vs. continuous airway positive pressure), demonstrating a good correlation index and fair concordance with the clinical score [55].

Pneumothorax (PNX) can be diagnosed using specific ultrasonographic signs, such as the absence of B-lines and of lung sliding and the presence of the stratosphere sign in M-mode imaging [56]. In adult patients, LUS showed high sensitivity and specificity, 91% and 98.2%, respectively [57]. Clinical experience in neonates and children is limited, but recent studies showed similar results in this population [58,59]. It should be noted that PNX detection in young children using LUS is more difficult than in adults and requires a certain degree of experience [59]. This is due to a faster respiratory rate, which complicates the detection of the lung point sign (passage point from normal lung to absent lung sliding). Furthermore, critically ill neonates are difficult to assess, as they cannot be easily mobilized from supine to prone position and vice versa. Moreover, PNX in children is usually limited in extension [60]. It follows that the adoption of two anterior longitudinal LUS scans, used for the extended fast ultrasound exam in adults, is inadequate to rule out a PNX in the paediatric emergency department [60].

A single case report described the presence of multiple hyperechoic artifacts among the intercostal fascial planes in a 17-year-old boy with pneumomediastinum (PNM) [61]. The integration of LUS with cervical ultrasonographic assessment could be useful to rule out PNM in children with acute chest pain. Nevertheless, this application needs further evidence to be validated.

Many other respiratory diseases have been found to show some pathological patterns with chest ultrasonography. For example, LUS has been employed for pulmonary tuberculosis assessment, showing higher sensitivity with respect to CXR in detecting consolidations, pleural effusion and enlarged lymph nodes [62,63]. In addition, LUS had a better inter-reader agreement for all the mentioned signs [62]. Other diseases that showed non-specific LUS findings are sickle cell disease [64], scoliosis [65], anomalous origin of left coronary artery from the pulmonary artery (ALCAPA) [66], acute asthma [67], interstitial lung diseases [68], neuromuscular diseases [69] and cystic fibrosis [70,71]. A case series of four patients with severe scoliosis showed that LUS could be useful to detect lung consolidation in these patients when CXR is hampered by costal deviations [65]. Patients affected by neuroendocrine cell hyperplasia of infancy (NEHI) showed an augmented B-line number, B-line density and pleural thickness [68]. An ultrasound pattern characterized by predominant B-lines, suggestive for interstitial involvement, has been described in children affected by cystic fibrosis [70]. A similar pattern, mostly characterized by multiple B-lines and pleural abnormalities, was observed during asthma exacerbation [67]. However, it is difficult to establish if those ultrasound alterations were due to asthma inflammation or underlying viral infections, major triggers of paediatric asthma.

These novel data about LUS application in miscellaneous pulmonary diseases are interesting and encourage further research. However, they should be regarded as preliminary results, and there is no current evidence supporting the use of LUS as a unique diagnostic tool in these disorders. All main findings characterizing the aforementioned disorders are summarized in Table 2.

## 6. LUS in COVID-19

The recent pandemic of the novel coronavirus SARS-Cov2 affects mainly the lungs, with variable degrees of interstitial inflammation and progressive lung consolidation [72]. The gold standard for COVID-19 pneumonia assessment in adult patients is a chest CT scan, but this imaging technique has been discouraged in children, who present generally with milder signs and symptoms [73,74]. CXR does not have high sensitivity or accuracy in diagnosing COVID-19 pneumonia [74]. LUS has been used widely in adult patients due to the ease of use and portability [75]. In a prospective observational study, the Soldati score was used to assess the aeration of symptomatic and asymptomatic children positive for SARS-CoV2, showing a score of 2–3 in patients with acute respiratory symptoms and a score of 2 in Multisystem Inflammatory Syndrome in Children (MIS-C) patients [76]. The most common sign was represented by little subpleural consolidations, <1 cm. A similar score was used to assess COVID-19 affected neonates, showing a good correlation with the clinical severity of the disease [77]. The most common pathological LUS findings in this group of patients were B-lines abnormalities. A Turkish multicentre study found that LUS had higher accuracy than CXR in detecting lung involvement in paediatric COVID-19 patients, using CT as the reference standard. In particular, specificity was 93.75% and sensibility 83.33%, with a PPV as high as 90.9% [78]. These data suggest that LUS could be used as a screening tool in children with probable SARS-CoV2 symptomatic infection, aiming to determine those with greater lung involvement and higher risk for respiratory support [12,79,80]. Nevertheless, LUS should not be considered a diagnostic tool for COVID-19, as ultrasonographic patterns are non-specific and shared with other respiratory viral infections, such as acute bronchiolitis [81]. In addition, children with a positive nasopharyngeal swab could show a normal LUS pattern, particularly if they are asymptomatic or have mild symptoms [82]. 

Therefore, LUS could be considered a valuable choice for COVID-19 follow-up in the paediatric population due to the lack of radiation exposure and fast performance [83,84]. CXR and CT should be limited to complicated cases, such as those with PNX [85,86]. However, LUS’ ease of use and tolerability could lead to its employment, even to screen patients with mild symptoms and few risk factors for COVID-19 progression. This could be of great interest, as there are limited data about post-COVID-19 symptoms and alterations, particularly in the paediatric population. On the other hand, it must be considered that minimal LUS alterations might be physiologic. It follows that LUS adoption for post-COVID-19 follow-up may raise concerns about overdiagnosis and overtreatment in otherwise healthy children.

## 7. Strengths and Limits of LUS in Children

Ultrasonography is a useful diagnostic tool for paediatricians as it is free of ionizing radiations and well tolerated by children of all ages. In addition, it is cheap, fast and can be performed in emergency departments or in Intensive Care Units at the patient’s bedside, sparing precious time and allowing assessment of severely ill patients who cannot be easily transported [87,88]. The advent and spread of POCUS examination reclassify LUS from a radiological imaging study to an extension of the objective evaluation made directly by the clinician [89,90]. In this context, the association of LUS with the clinical presentation allows it to reach the best possible sensitivity and specificity, avoiding more invasive and time-consuming tools, such as a CT scan.

In the present context of the COVID-19 pandemic, the adoption of LUS to screen and assess suspected and confirmed cases helps physicians to limit viral spreading, as the patient can be directly assessed inside the isolation ward and the ultrasound machine can be easily sterilized [91].

Obviously, ultrasonography is not an ideal diagnostic tool per se. First, ultrasounds allow only one anatomical slice to be scanned at a time, and a global reconstruction of the examined body part is currently not possible in clinical practice [92,93]. Secondly, LUS is limited to superficial surfaces of the lung, thus lesions that do not reach the pleura are not detected [94]. Moreover, bony structures (ribs, clavicles and scapulae) block ultrasounds, hiding lesions underneath [94]. It should be considered that children, and in particular neonates, have a less calcified thoracic cage with respect to adults, facilitating LUS assessment in these patients [22]. 

Another limit of LUS is its intrinsic operator-dependency [95]. In fact, Gravel et al. [9] demonstrated that interrater reliability (IRR) of lung POCUS in children presenting with respiratory symptoms associated with pneumonia was moderate [9]. There was only fair agreement for left sided zones compared to the right ones. These results should be discussed, taking into consideration previous data for specific clinical examination findings and chest X-ray, whose interrater reliability ranges from minimal to moderate [10,96,97]. Other studies found very high IRR for LUS, even comparing operators with different experience [53,62,98,99]. Indeed, many authors suggested that LUS can be easily performed with good results in terms of diagnostic accuracy even by novices with short training programs. A LUS training protocol with both theorical and practical sessions showed a significant improvement in diagnostic sensitivity during the first four months of the study [100]. The agreement between study physicians and expert readers increased gradually, peaking at six months of independent scanning after a five month period of supervised training [100]. In another prospective observational study, novel operators performing LUS showed greater sensitivity and less specificity detecting pneumonia compared to expert ones [38]. Agreement was very good among experts, in particular for the identification of consolidation patterns. On the contrary, a recent meta-analysis including 3353 participants found greater sensitivity for expert operators and greater specificity for novel ones [101]. Taken together, these findings highlight a difference in accuracy for the identification of pathological findings on LUS between experienced and novel operators, suggesting the need for a standardized training program.

LUS advantages and limits are summarized in Table 3.

## 8. Future Perspectives

The interest in LUS has risen steadily and rapidly in the last several years, as highlighted by the great number of recent papers present in the scientific literature about this issue. The safety and affordability of this imaging technique are attractive characteristics, especially for paediatric patients who are more vulnerable to ionizing radiations. The use of LUS as a bedside tool, together with a detailed medical history and clinical assessment, might decrease the need for chest radiographs and ameliorate the management and treatment of children affected by respiratory diseases. In particular, LUS can be used during the follow-up of patients affected by pneumonia, RDS, bronchiolitis and pleural effusion to monitor the regression of the pathological process. However, a few points need to be addressed by future studies in order to improve its use in clinical practice. First of all, ultrasonographic findings of uncertain significance, such as subpleural consolidation of less than 1 cm and multiple B-lines, need to be better characterized [37]. These alterations are common in children with respiratory tract infections and could prompt paediatricians to over-prescribe antibiotic treatment [102]. Indeed, the aetiology of those small subpleural consolidations is yet unknown. Large multicentre observational studies are needed to reach this important goal. Secondly, the training protocol should be standardized, improving the interpretation of study results and reducing the heterogeneity for diagnostic accuracy [101]. Once standardized, POCUS lung examination could become a valid screening tool to support clinical diagnosis in children with suspected pneumonia and other respiratory diseases. This could help physicians to follow paediatric guidelines and to reserve radiological examinations only for uncertain cases. Finally, the validation of LUS scores and diagnostic power could represent a real revolution for the management of children affected by lung diseases in developing countries, as ultrasound machines and training are cheaper than X-rays or CT scans [103]. However, further longitudinal observational studies with larger samples and better patient characterization are needed to implement the use of this imaging technique in clinical practice. 

## Figures and Tables

**Table 1:** LUS main findings in neonatal lung diseases. LUS: Lung Ultrasounds.

Neonatal Lung Diseases	Main LUS Findings
Transient tachypnoea of the newborn	■ Dense confluent B-lines ■ Double-lung point ■ Spared lung areas ■ No consolidations
Neonatal respiratory distress syndrome	■ Dense confluent B-lines ■ Whole lung involved ■ Interspersed consolidations
Meconium aspiration syndrome	■ Diffused lung consolidations ■ B-lines ■ Pleural irregularities
Congenital pulmonary malformations	■ Cystic lesions ■ Tissue-like lesions

**Table 2:** LUS main findings in miscellaneous lung diseases. LUS: Lung Ultrasounds; NEHI: Neuroendocrine Cell Hyperplasia of Infancy.

Pulmonary Diseases	Main LUS Findings
Bronchiolitis	■ Consolidations ■ Pleural irregularities ■ Diffused B-lines
Pneumothorax	■ Absent lung-sliding ■ No B-lines ■ Physiologic A-lines pattern ■ Stratosphere sign in M-mode
Pneumomediastinum	■ Multiple hyperechoic artifacts in the intercostal fascial planes
Tuberculosis	■ Consolidations ■ Pleural effusion ■ Enlarged lymph nodes
Severe scoliosis	■ LUS helps to detect consolidations hidden by costal deformities
NEHI	■ B-lines ■ Thickened pleura
Cystic fibrosis	■ Diffused B-lines

**Table 3:** Summary of LUS advantages and limits. LUS: Lung Ultrasound.

LUS Advantages	LUS Limits
■ Radiation free ■ Cheap ■ Bedside examination ■ Relatively easy to use ■ Fast exam, ideal for emergency assessment ■ High sensitivity and specificity when associate with the clinical presentation	■ Focused anatomical visualization ■ Cannot detect lesions that do not reach the pleura ■ Ultrasounds are blocked by bone structures ■ Low resolution in obese patients ■ Operator dependent

## Data Availability

According to the typology of paper (narrative review) there is no data storage available.
